# Impact of E-Cigarettes on Fetal and Neonatal Lung Development: The Influence of Oxidative Stress and Inflammation

**DOI:** 10.3390/antiox14030262

**Published:** 2025-02-25

**Authors:** Antonella Gambadauro, Francesca Galletta, Beatrice Andrenacci, Simone Foti Randazzese, Maria Francesca Patria, Sara Manti

**Affiliations:** 1 Pediatric Unit, Department of Human Pathology in Adult and Developmental Age “Gaetano Barresi”, University of Messina, 98124 Messina, Italy; francygall.92@gmail.com (F.G.); sara.manti@unime.it (S.M.); 2 S.C. Pneumoinfettivologia Pediatrica, Fondazione IRCCS Ca’ Granda Ospedale Maggiore Policlinico, 20122 Milan, Italy; beatrice.andrenacci@policlinico.mi.it (B.A.); francesca.patria@policlinico.mi.it (M.F.P.)

**Keywords:** air pollution, asthma, COPD, e-cigarette, lung development, neonates, oxidative stress, pregnancy

## Abstract

Electronic cigarettes (e-cigs) recently increased their popularity as “safer” alternatives to traditional tobacco smoking, including among pregnant women. However, the effect of e-cig exposure on fetal and neonatal developing lungs remains poorly investigated. In this review, we analysed the impact of e-cig aerosol components (e.g., nicotine, solvents, and flavouring agents) on respiratory system development. We particularly emphasized the role of e-cig-related oxidative stress and inflammation on lung impairment. Nicotine contained in e-cigs can impair lung development at anatomical and molecular levels. Solvents and flavours induce inflammation and oxidative stress and contribute to compromising neonatal lung function. Studies suggest that prenatal e-cig aerosol exposure may increase the risk of future development of respiratory diseases in offspring, such as asthma and chronic obstructive pulmonary disease (COPD). Preventive strategies, such as smoking cessation programs and antioxidant supplementation, may be essential for safeguarding respiratory health. There is an urgent need to explore the safety profile and potential risks of e-cigs, especially considering the limited studies in humans. This review highlights the necessity of regulating e-cig use during pregnancy and promoting awareness of its potential consequences on fetal and neonatal development.

## 1. Introduction

In the last decade, researchers warned the global population about indoor and outdoor pollution’s harmful effects on respiratory health in adults and children [1]. Despite the increased knowledge about the deleterious impact of nicotine and other additives on the lungs, new electronic nicotine delivery systems (ENDSs) became popular among consumers and often suggested for smoking cessation and reported as safer than tobacco cigarettes [2,3]. Electronic cigarettes (e-cigs) are one of the most popular ENDSs in commerce. These devices may also not contain nicotine, and, in this case, they can be classified as electronic non-nicotine delivery systems (ENNDSs) [4]. E-cigs are non-combustible systems comprising a battery, a microprocessor, a start button, a heating part, and a liquid reservoir [5]. The solution in the reservoir is vaporized during use, producing an aerosol (or vapour) of highly concentrated droplets mainly formed by sub-micrometre particles [6]. In addition to the potential presence of nicotine, the liquid solution may contain a wide variety of flavours, solvents (e.g., propylene glycol (PG), vegetable glycerine (VG), glycerol), and preservatives [7,8]. Trace metals and additional impurities (e.g., polyaromatic hydrocarbons, aldehydes, and acrolein) have also been detected in the aerosol [9,10,11].

ENDS use during pregnancy was reported in percentage rates ranging from 0.6% to 15% in different analyses [12,13,14,15]. An online national survey conducted in the United States (USA) on 445 pregnant women reported that 6% and 7% used only tobacco cigarettes or only e-cigs, respectively, while 9% used both tobacco cigarettes and e-cigs [16]. These findings must be read considering the general perception that e-cigs are a healthier alternative to traditional cigarettes and may assist with smoking cessation during pregnancy [17].

The fetal lungs are organs sensitive to toxins, which can alter their normal neonatal development and predispose them to respiratory diseases later in life [18]. Few data are available on the short- and long-term influences of e-cigs on respiratory development during the fetal and neonatal period due to the lack of safety studies [19]. The latest evidence suggests that nicotine and chemical products generated from e-cigs during the perinatal period could promote the activation of inflammation and the production of oxidative compounds, altering lung development and increasing the incidence of respiratory diseases in offspring [20,21].

In this review, we summarized and critically analysed the existing literature about the impact of e-cigs on fetal and neonatal lung development. There is an urgent need to determine if these electronic devices could be safe for offspring or potentially related to functional and structural lung impairment. By combining the key terms “e-cigarettes” OR “e-cigs” AND “lung development” OR “airway” OR “lung” AND “pregnancy” OR “neonates” in a computerized search of PubMed limited to the last 10 years, we provided a comprehensive overview of the literature, based on a critical evaluation without standardized methodologies or statistical analyses.

## 2. Stages of Normal Lung Development

Lung development is a sequential, multistep process that begins prenatally and continues postnatally, reaching completion in early adulthood [22,23]. This extended timeline makes the respiratory system vulnerable to prenatal and postnatal influences, including prematurity, low birth weight, maternal and personal nutrition, exposure to smoking, environmental pollutants, and early-life infections. These factors interact with individual genetic and epigenetic backgrounds to shape respiratory function [22]. Lung function at birth is a key determinant of lung health across the lifespan, and supporting optimal lung development during early life is crucial to mitigate the natural decline in respiratory function with ageing [22].

**Figure 1:**
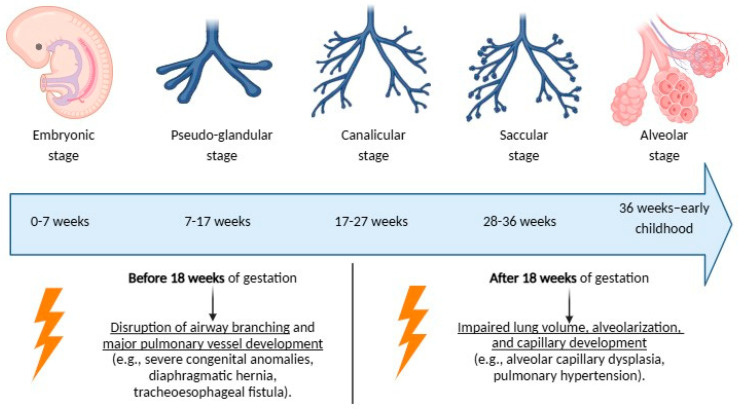
Stages of normal lung development and impact of adverse exposures.

Prenatal lung development is classically divided into five stages from 3 to 36 weeks of gestation: the embryonic, pseudo-glandular, canalicular, saccular, and alveolar stages (Figure 1). Each phase represents a critical window during which specific structural and functional components of the lungs are established [23].

During the embryonic stage (0–7 weeks), the first rudiment of the respiratory system, which is called the laryngotracheal groove, appears on the ventral wall of the anterior foregut, extending caudally into the respiratory diverticulum [22]. The trachea separates from the oesophagus via tracheoesophageal septa, and the “branching morphogenesis” begins [22,24]. This process first generates the two main bronchi and progresses to lobar and segmental bronchi until the development of 18 major lobules, laying the groundwork for the airway tree. Simultaneously, angiogenesis begins around the developing respiratory structures, initiating the formation of the pulmonary vasculature [22].

In the pseudo-glandular stage (7–17 weeks), vasculogenesis and branching morphogenesis advance rapidly, forming terminal bronchioles by 14 weeks of gestation. By the end of this stage, approximately 70% of the airway tree is complete [22]. Airway epithelial cells also begin to differentiate, with columnar cells appearing in the proximal airways and cuboidal cells in the distal regions [22]. Fetal breathing movements and airway contractions play a pivotal role in stimulating epithelial proliferation, differentiation, and early surfactant production. These mechanical stimuli stretch the lung tissue, promoting cellular development through pathways like serotonin upregulation [25,26].

The canalicular stage (17–27 weeks) is characterized by the development of respiratory bronchioles, alveolar ducts, and primitive alveoli, establishing the functional units of the lungs known as “pulmonary acini” [24]. During this stage, type I and type II pneumocytes differentiate, with type II cells beginning to produce surfactant precursors, known as lamellar bodies, by 24 weeks of gestation [24]. Early surfactant production reduces surface tension within the lungs, stabilizes alveoli during inflation, and facilitates effective breathing [24]. At the end of this stage, the formation of the alveolar–capillary barrier and the onset of gas exchange capability makes it possible for a preterm newborn to survive [24].

Branching morphogenesis concludes in the saccular stage (28–36 weeks of gestational age), with saccules developing from pulmonary acini, accompanied by a significant increase in gas exchange potential and surfactant production [24].

The alveolar stage (36 weeks–early childhood) begins shortly before birth, and it involves the development of new alveoli through secondary septation of terminal saccules [22]. The alveolar number increases rapidly during the first 2–4 years of life, reaching an estimated 300–800 million at around 8 years of age; then, alveolarization slows, though alveolar size continues to grow until adolescence, with interindividual variability and slight gender differences [27,28]. Recent advances in imaging techniques, such as high-resolution tomography and helium-3 magnetic resonance imaging, suggest that alveolarization may persist into adulthood, though the exact timeline and molecular mechanisms remain under investigation [29,30,31]. During this period airways expand proportionally in diameter and length, maintaining a constant length-to-diameter ratio [32]. Concurrently, the pulmonary microvasculature undergoes remodelling, with the double-layered network fusing into a single-layered structure through capillary fusion and thinning of the alveolar septa. The resulting alveolar–capillary membrane, composed of alveolar epithelial cells, capillary endothelial cells, and their shared basement membrane, becomes the key site for efficient respiratory gas exchange [22,24].

The respiratory system remains highly sensitive to environmental and genetic factors throughout its development, with long-term outcomes depending on the timing and nature of exposures during critical windows of susceptibility (Figure 1) [33].

Adverse exposures occurring before 18 weeks of gestation may disrupt airway branching and major pulmonary vessel development, resulting in severe congenital anomalies, such as pulmonary agenesis or aplasia, which are often incompatible with life, or conditions like diaphragmatic hernia with pulmonary hypoplasia and tracheoesophageal fistula, which carry significant morbidity and mortality [24].

Instead, later exposures, occurring after 18 weeks of gestation, generally affect lung volume, alveolarization, and capillary development. For example, alveolar capillary dysplasia (ACD) may result from insults during this stage, characterized by reduced capillary density, misaligned pulmonary veins, and thickened alveolar septa, leading to pulmonary hypertension [34]. Additionally, disturbances in the normal pattern of angiogenesis and alveolar maturation can impair gas exchange efficiency and lung compliance, often leading to chronic respiratory complications [35].

Finally, adverse exposures after birth, such as air pollution, second-hand smoke, or recurrent respiratory infections, might impair lung growth and the development of pulmonary microvasculature [22]. Young children are particularly vulnerable to inhaled pollutants due to their relatively higher airway flow rates and greater deposition of small particles than adults [36]. These vulnerabilities highlight the importance of protective measures and early interventions to preserve respiratory health and promote optimal lung development.

## 3. Are E-Cigarettes Safe?

### 3.1. Impact on Lung Development

In recent years, especially among young people, the interest in e-cigs has increased. Furthermore, their use is intensifying in pregnant women due to the perception of their safety compared to tobacco cigarettes [17,37]. The reservoir of these devices contains e-liquid, which is converted into aerosols by a heating process. E-liquid is a mixture of water, different nicotine levels, solvents, flavours, preservatives, and other potential additives [7,8] (Table 1).

Nearly 40% of pregnant women believe that e-cigs do not contain nicotine [12]. Nicotine is a dinitrogen alkaloid extracted from tobacco plants. It is a parasympathomimetic stimulant and a dependence-forming constituent of e-cigs and tobacco cigarettes [38]. In pregnant women, nicotine crosses the placenta and reaches high concentrations in fetal blood and amniotic fluid [39,40]. During breastfeeding, nicotine levels are higher in breast milk than in maternal plasma [41,42]. No amount of nicotine is known to be safe during pregnancy [43]. ENDSs contain diverse nicotine concentrations, and previous studies conducted on animals and humans reported similar or higher nicotine levels in e-cigs compared to tobacco cigarettes [44,45]. In a study conducted on rodents, nicotine concentrations were eight times higher after e-cig exposure (JUUL type) compared to tobacco cigarette exposure [44]. A study conducted on 13 healthy adult e-cig users who took 15 puffs from their usual e-cigs revealed that nicotine levels were comparable to or higher than classic tobacco cigarettes [45]. These results are relevant for understanding the potential impact of e-cigs during pregnancy. However, the most important limitation of these findings is the different nicotine levels in the diverse formulations of e-cigs studied.

Few data are reported in the literature about the effects of nicotine contained in e-cigs on human lung development. Most studies have been conducted on animal models and not exclusively during the in utero period. However, they can assist in comprehending the effect of maternal e-cig use and exposure during the critical first few weeks of life on the lung development of offspring. Ozekin et al. tested the impact of maternal vaping on fetal lung development in mice by low–moderate daily exposure to 2.4% nicotine vapour during the entire gestational period [46]. They specifically focused on the mouse E18.5 timepoint, considered as the last day of the embryonic stage, and reported that maternally vaped wildtype mouse lungs have smaller airspaces compared to room-air-exposed mice [46]. This finding was consistent with the disruption of the saccular stage of lung development in the same model [46]. A separate study analysed the impact of the daily inhalation of 36 mg/mL of nicotine cinnamon-flavoured e-cig aerosols during the preconception period (12 days before mating) and during gestation in a murine model [47]. Lung morphometry evaluations of preconception e-cig-exposed offspring reported a notably increased tissue fraction at birth related to a delayed thinning of the lung epithelium during the saccular stage [47]. Both of these studies showed wide gene expression changes in maternally vaped embryos dominated by the downregulation of developmental signalling pathways such as Notch and Wnt [46,47]. Notch and Wnt are pivotal regulators of lung development. Notch regulates ciliated versus secretory cell expression in the developing airways, and ciliated cell loss associated with downregulated Notch expression was reported in smokers and in patients with chronic obstructive pulmonary disease (COPD) [48,49]. The Wnt signalling pathway is essential in the saccular stage, promoting the proliferation and differentiation of alveolar epithelial cells, stem cell maintenance, and lung branching morphogenesis [50]. These findings confirm, at both the anatomical and molecular levels, that the nicotine contained in e-cigs impairs fetal lung development. Another study on mice compared e-cig aerosol exposure during pregnancy with and without nicotine to room-air controls at gestational day (GD) 21, at postnatal day (PND) 4, and at PND 10 [19]. The group exposed to e-cig aerosol vaping containing nicotine reported decreased lung size, increased free space within the lung parenchyma, and decreased alveolar septation compared to the other two groups at PND 4 [19]. In humans, the equivalent developmental stage of murine PND 4 occurs in the alveolar stage. These results suggest the specific role of nicotine contained in e-cig vapours (without the confounding factor of flavours) in determining an emphysematous phenotype with larger and fewer distal air spaces in neonatal mice [19]. Postnatal lung growth and weight gain were also reduced in neonatal mice after exposure to e-cigs containing 1.8% nicotine and PG for the first ten days of life compared to room air controls [51]. Based on these results, we can conclude that nicotine contained in e-cigs can affect lung development by altering airway growth, especially during the embryonic, saccular, and alveolar stages, inducing a final emphysematous phenotype and low lung size at birth.

Mucociliary clearance is essential to defend against the organisms that cause respiratory infections or the inhalation of irritants [52]. A bullfrog palate paradigm (a well-established model used to study mucociliary clearance) confirmed a modest dampening effect of e-cig aerosol on mucociliary clearance [53]. The impact on the mucociliary function seems principally related to the nicotine contained in e-cigs. In a study conducted on a murine model, chronic (3 weeks), daily, 20 min-exposure to nicotine and PG (e-cigs) slowed mucociliary clearance in murine lungs. Conversely, exposure to PG alone did not impact mucociliary clearance [54]. A study conducted in both in vitro human bronchial epithelial cells obtained from never-smoking individuals and in vivo sheep bronchial epithelial cells showed that nicotine-containing e-cig vapour induced mucociliary dysfunction by acting on TRPA1 (transient receptor potential ankyrin 1) [55]. TRPA1 is a nicotine-sensitive receptor that belongs to the TRP ion channel family, and it is extensively distributed in the cellular membranes of humans and mammals [56]. The impairment in mucociliary clearance, by altering mucus viscoelasticity, increasing mucus/mucin concentrations, and damaging mucociliary transport, may affect the protection of the airway and increase infectious diseases in neonates and infants born to vaping women. Studies on the neonatal period are needed to confirm this hypothesis.

The two solvents most frequently included in e-cigs are PG and VG. Both PG and VG were initially considered innocuous components of e-cigs. However, recent studies have analysed their impact on lung development. During vaping, the thermal decomposition of PG generates acetone, acetaldehyde, and formaldehyde, while the thermal decomposition of VG produces principally acrolein and formaldehyde [57]. Among these substances, acetaldehyde and formaldehyde are classified as carcinogens, while acrolein is considered a human carcinogen able to irritate the upper respiratory tract after inhalation [58]. An in vivo study on human bronchial epithelial cells (HBECs) obtained from never-smoking individuals revealed that a one-week exposure to VG and PG/VG-containing e-cigs (in the absence of nicotine and flavours) decreased the function of cystic fibrosis transmembrane conductance regulator (CFTR), an anion channel important for mucus hydration [59]. In volunteers exposed to VG-containing e-cigs, the reduction in CFTR function was associated with high inflammatory biomarkers, such as Interleukin-6 (IL-6), IL-8, matrix metalloproteinase-9 (MMP9) mRNAs, MMP-9 activity, and mucin 5AC (MUC5AC) expression levels [59]. These modifications induced a reduction in ciliary beating and increased mucus concentration as a possible consequence of goblet cell hyperplasia [60]. Exposure during pregnancy to PG/VG-containing e-cig vapour with and without nicotine was tested in murine neonates at 5 months of age [61]. Female mice exposed to PG/VG-containing e-cig vapour with and without nicotine reported goblet cell hyperplasia. The group exposed only to solvents (without nicotine) showed increased lung and alveolar stiffness (both genders) and decreased lung compliance (only female mice) [61]. This last finding could be the result of increased extracellular matrix (ECM) deposition, which was reported as a cause of PG/VG-containing e-cig aerosol exposure (without nicotine) during pregnancy [62].

Few studies consider the impact of flavours contained in e-cigs during pregnancy, and most of them consider the preferences of pregnant women. Among flavouring agents, fruit and mint are the most used in this population [8]. However, the impact of specific flavours on lung development in the fetal and neonatal periods was not critically analysed in previous research. Each flavouring agent may potentially have different inflammatory and toxic effects on lung morphogenesis and function. Moreover, e-cigs containing several flavours are more toxic and induce a higher inflammatory response than single flavours [63]. Maltol, ethyl maltol, ethyl vanillin, vanillin, and furaneol have been reported as the most cytotoxic flavouring agents in e-cigs [64]. By considering the respiratory system, cinnamaldehyde, 2-methoxycinnamaldehyde, O-vanillin, and pentanedione seem to have cytotoxic effects on airways [65]. Further studies are needed to analyse the role of different flavours on the developmental lungs.

**Table 1:** An overview of the main harmful effects of e-cigarette compounds on lung development.

Chemical Compound	Harmful Effect	References
Nicotine	Crosses the placenta, accumulates in fetal blood and amniotic fluid, disrupts lung development (embryonic, saccular, and alveolar stages), reduces lung size, induces the emphysematous phenotype, impairs mucociliary clearance.	[19,39,40,46,47,48,49,50,51,53,54,55]
PG/VG	Their thermal decomposition generates carcinogens and irritants (PG produces acetone, acetaldehyde, and formaldehyde, while VG produces acrolein and formaldehyde); PG/VG exposure reduces CFTR function and increases inflammatory biomarkers, leading to lower ciliary beating and higher mucus concentration; PG/VG exposure decreases lung compliance, increases alveolar stiffness, and induces extracellular matrix deposition.	[57,58,59,60,61,62]
Flavouring agents	Some flavours (e.g., cinnamaldehyde, O-vanillin, pentanedione) have cytotoxic and inflammatory effects on airways.	[63,64,65]
PM_2.5_ and UFPs	Oxidative stress by generating ROS; activation of inflammatory pathways.	[66]

CFTR = cystic fibrosis transmembrane conductance regulator; PG = propylene glycol; VG = vegetable glycerine; UFPs = ultrafine particles; PM_2.5_ = particulate matter 2.5.

### 3.2. The Role of Oxidative Stress

**Figure 2:**
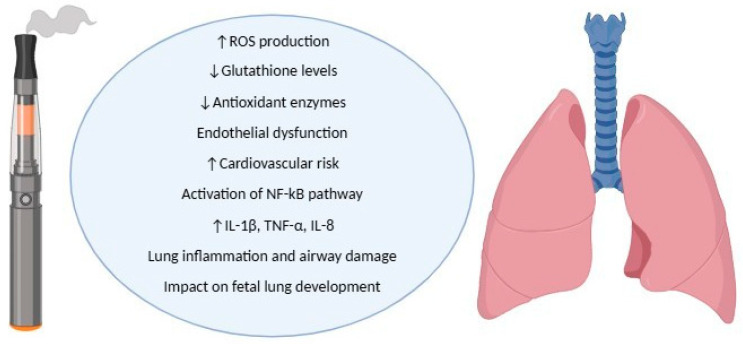
The role of e-cig-induced oxidative stress on the airways.

Oxidative stress has a central role in the development and maintenance of numerous lung diseases [67]. E-cigs can directly or indirectly induce oxidative damage to human lungs due to the presence of reactive chemical substances in their aerosols, the activation of pro-inflammatory compounds, and the modulation of intracellular pro-inflammatory pathways. E-cig aerosols contain diverse toxic compounds (e.g., formaldehyde, acetaldehyde, and acrolein), reactive oxygen species (ROS), heavy metals, flavours, and propylene oxide, which is derived from PG heating [68]. It was reported that e-cig vapour contains 7 × 10^11^ free radicals per puff, eliciting an important increase in oxidative stress [69]. Moreover, several studies have described the presence of significant amounts of fine (PM_2.5_) and ultrafine particles (UFPs) in e-cig aerosol [66] (Table 1). Nicotine, PG, and VG play a pivotal role in determining the cytotoxic and pro-inflammatory effects of these aerosols. PG and VG induce cytotoxic effects depending on puff number, while nicotine seems protective against cellular toxicity [66]. Lung inflammation is secondary to the activation of the pro-inflammatory NF-kB pathway, which induces IL-1β and tumour necrosis factor (TNF)-α production [66]. Interestingly, a study on murine models reported an abnormal inflammatory environment in the airways of both mothers and offspring after e-cig exposure during pregnancy [70]. In that research, TNF-α was increased in the lungs of both the mothers and offspring; and IL-1β was increased only in the mothers [70]. An in vivo study conducted on human bronchial airway epithelial cells (H292) and human fetal lung fibroblasts (HFL1) showed that the inflammatory response and the stress phenotype depended on nicotine content and flavour type [71]. For example, cinnamon-flavoured e-liquid produced a higher IL-8 response than cigarette smoke extract [71]. The exposure of wild-type mice to e-cig aerosols decreased lung glutathione levels, increasing oxidative airway damage [71]. A recent study on rat lung tissues confirmed that exposure to e-cig aerosols reduced the activity of antioxidant compounds, such as superoxide dismutase, catalase, and glutathione peroxidase [72]. Pulmonary microvascular endothelial cells can also be the target of oxidative stress damage. Exposure to e-cig vapour contributes to vascular endothelial dysfunction in the cardio-respiratory system, predisposing to hypertension and atherosclerosis [73,74]. E-cig vapour exposure (with or without nicotine) increases the expression of NADPH oxidase (NOX), an enzymatic source for ROS during inflammation [75]. Furthermore, it induces endothelial nitric oxide synthase (eNOS) uncoupling, increasing superoxide generation and peroxynitrite production, as well as tetrahydrobiopterin (BH4) reduction [75]. All these changes decrease nitric oxide (NO) synthesis and availability, inducing endothelial dysfunction and long-term cardio-respiratory system impairment [75]. These findings are useful in improving our understanding of the potential oxidative harm produced by e-cigs on the airways (Figure 2). However, studies on fetal and neonatal lungs may be helpful in understanding the effects of e-cig-induced oxidative stress during pregnancy.

### 3.3. Long-Term Effects on Respiratory Health

Maternal smoking during pregnancy is related to a twofold increase in the risk of wheezing and asthma in offspring [76,77]. Several studies reported that this increased risk seems to be associated with nicotine alone during perinatal smoke exposure [18]. During gestation, the use of devices containing tobacco, such as heated tobacco products (HTPs), was associated with an increased risk of onset of allergic diseases (e.g., asthma, allergic rhino/conjunctivitis, atopic dermatitis) in offspring [78]. However, few studies have been conducted on e-cigs, and most of the literature is based on the effect of tobacco cigarettes. Nicotine is the primary agent responsible for impaired lung development. Maternal smoking exposure is associated with reduced forced expiratory flows, decreased passive expiratory compliance, higher hospitalization rates for respiratory infections, and increased prevalence of childhood wheezing and asthma [20]. A study conducted in a murine model analysed e-cig aerosol exposure (with and without nicotine) during pregnancy [19]. The findings of that research revealed increased respiratory resistance and reduced compliance in mice exposed to nicotine-containing e-cigs compared to controls [19]. Infants and children exposed to passive smoking during pregnancy showed higher airway resistance and increased rates of persistent wheezing [79,80]. Adolescents using e-cigs are more likely to have chronic cough, bronchitis, and asthma [81]. Asthmatic children and adolescents exposed to second-hand e-cig smoke at home have more daily respiratory symptoms compared to unexposed controls [82]. In adults, an association between e-cigs and the development of asthma or COPD was found, especially in non-smokers [83]. E-cig vapour may also suppress the immune response to viral and bacterial infections, increasing the host susceptibility by impairing pulmonary anti-microbial defences [69]. The literature on the long-term effects of e-cigs on respiratory health is still lacking. However, asthma and COPD seem to be the two conditions more associated with e-cig exposure.

## 4. Prevention Strategies

The adoption of effective prevention strategies represents a pivotal step in mitigating the adverse effects of e-cig use on fetal lung development. These strategies are essential not only for safeguarding the fetus’s health but also for enhancing maternal health outcomes [84]. Prevention strategies require a multifaceted approach that integrates smoking cessation programs, legislative efforts, dietary interventions, and the active involvement of healthcare providers [20].

Smoking cessation remains the cornerstone of preventing the harmful effects of e-cigs on both the mother and fetus [84]. Despite the well-documented risks to lung development and overall fetal health, a significant number of pregnant women continue smoking due to addiction, limited access to support, or socioeconomic barriers [85,86,87]. Successful cessation strategies, therefore, play a critical role in reducing these risks [84]. The effectiveness of various smoking cessation interventions has been extensively evaluated. Behavioural counselling is one of the most effective interventions for smoking cessation during pregnancy. Evidence from systematic reviews demonstrated that intensive psychosocial support, including motivational interviewing and behavioural therapies, can significantly increase cessation rates. Counselling programs tailored to the unique needs of pregnant women are especially effective, particularly when delivered through multiple sessions by trained professionals [85,88]. Physical activity programs have been explored as adjunct strategies to traditional behavioural support [89]. Although their effectiveness in promoting smoking cessation during pregnancy remains uncertain, physical activity contributes to overall health benefits and has been shown to reduce smoking urges in non-pregnant individuals. These findings suggest the potential for further research of these interventions in future studies [89]. Emerging technologies such as Artificial Intelligence (AI) have introduced innovative approaches to smoking cessation [90]. AI-powered tools can provide real-time, personalized support, analyse individual behavioural patterns, and predict relapse risks [90]. For instance, mobile applications can deliver motivational messages, monitor smoking habits, and recommend tailored coping strategies. Virtual support groups facilitated by AI platforms have also proven effective in fostering community engagement [90]. Financial incentives have been identified as another effective tool in promoting smoking cessation among pregnant individuals, particularly those from disadvantaged socioeconomic backgrounds. Studies reveal that offering monetary or voucher-based rewards for achieving abstinence significantly increases cessation rates and reduces the economic burden of smoking-related complications [91,92,93].

Healthcare providers, particularly midwives and obstetricians, play a critical role in smoking cessation efforts during pregnancy. Routine screening for tobacco use during prenatal visits, combined with timely referrals to cessation resources, is essential. However, challenges such as time limitations and resource restrictions often hinder the consistent implementation of these interventions [88,94,95,96]. Overall, integrating evidence-based strategies like counselling, financial incentives, and healthcare provider engagement into prenatal care can significantly enhance smoking cessation rates during pregnancy, improving both maternal and fetal outcomes [95]. Additionally, systematic reviews and population-based studies have highlighted both the potential benefits and risks associated with Nicotine Replacement Therapy (NRT) and other pharmacological interventions, such as bupropion and varenicline, for smoking cessation during pregnancy [97,98,99,100,101]. NRT is commonly used to support smoking cessation in the general population and is sometimes prescribed during pregnancy to mitigate nicotine withdrawal symptoms [98]. Studies have demonstrated that NRT can increase the likelihood of smoking cessation during pregnancy when combined with behavioural support. A Cochrane review showed that NRT improves cessation rates by approximately 37% compared to placebos, although with low-certainty evidence [98,99]. Faster-acting formulations, such as nicotine gum or tablets, have been suggested as potentially safer options compared to nicotine patches, which provide continuous nicotine delivery [102]. However, concerns persist about potential risks, including increased rates of infant colic and attention-deficit/hyperactivity disorder (ADHD) in children exposed to nicotine during gestation [97,103]. Consequently, the use of NRT during pregnancy remains controversial due to its potential effects on fetal development [98]. Bupropion, an atypical antidepressant, and varenicline, a partial nicotine receptor agonist, are other pharmacological options that have been studied for smoking cessation during pregnancy [100]. Evidence suggests that bupropion can be effective without significantly increasing the risk of adverse perinatal outcomes, such as preterm birth or congenital anomalies [100,101]. Varenicline, while effective in the general population, has limited data on its safety in pregnancy, and guidelines generally recommend caution due to insufficient evidence [100,101]. To date, no information is currently available regarding the effects of either bupropion or varenicline on fetal lung development. Overall, while pharmacological interventions such as NRT, bupropion, and varenicline may support smoking cessation during pregnancy, their use should be carefully balanced against potential risks [100]. Healthcare providers should adopt a patient-centred approach, tailoring pharmacological strategies to individual needs and circumstances, and should ensure that such treatments are complemented by behavioural and psychosocial support [99,101,104]. Further research is needed to provide clearer evidence regarding the safety and efficacy of these interventions in pregnancy.

Legislative measures could have a pivotal role in promoting smoking cessation during pregnancy. Policies that restrict the sale of e-cigs prohibit their use in public spaces and mandate clear labelling of nicotine content can discourage use. Additionally, public health campaigns focused on raising awareness about the risks of e-cigs, particularly during pregnancy, can enhance the effectiveness of these legislative actions by educating individuals to make healthier choices [105].

The use of antioxidants during pregnancy for smoking mothers has shown promise in mitigating respiratory issues in offspring. Vitamin C has been extensively studied for its protective effects against oxidative stress induced by maternal smoking [106,107,108]. A randomized trial demonstrated that daily supplementation with 500 mg of vitamin C during pregnancy improves newborn pulmonary function, reduces wheezing, and prevents DNA methylation changes associated with maternal smoking [109]. These changes suggest that vitamin C mitigates the epigenetic alterations linked to reduced lung development and long-term respiratory risks.

Vitamin E, another powerful antioxidant, has been shown to synergize with vitamin C to contrast oxidative stress in placental tissues, thereby protecting fetal development from the adverse effects of nicotine [110]. Similarly, N-acetylcysteine (NAC), by enhancing glutathione synthesis, has demonstrated protective roles in reducing oxidative stress and supporting placental function [110]. Meanwhile, L-carnitine has shown efficacy in reducing inflammation and oxidative damage in animal studies, improving birth weight and partially normalizing inflammatory markers in offspring exposed to maternal smoke [111].

## 5. Conclusions

E-cigs are significant modifiable risk factors for short- and long-term respiratory damage in fetal and neonatal airways. Nicotine and other compounds are responsible for anatomical and molecular modifications of the lungs during pregnancy and the perinatal period. Oxidative stress has a pivotal role in the activation of inflammatory damage in the respiratory system. Studies in humans are urgently needed to confirm the sequelae of the exposure on the developing airways.

**Figure 3:**
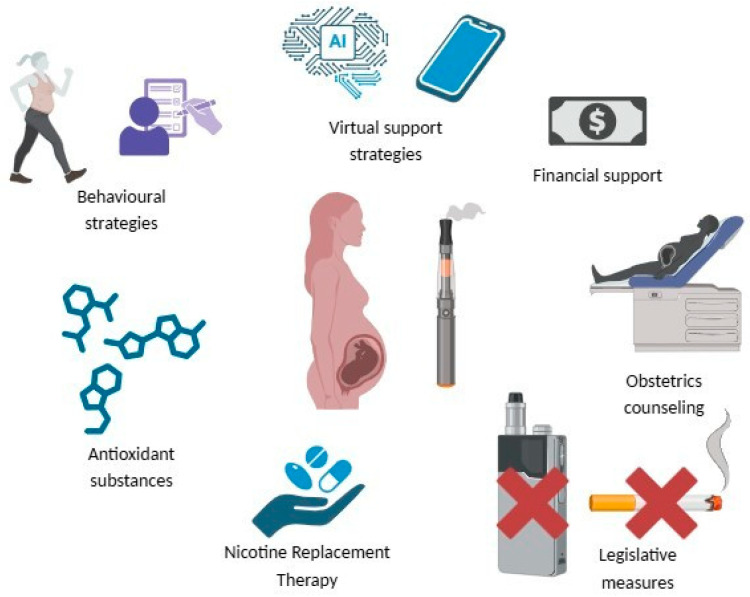
Multiple integrated prevention strategies to influence e-cig use during pregnancy.

A comprehensive prevention strategy should integrate multiple interventions to address the diverse factors influencing e-cig use during pregnancy (Figure 3). Legislative measures and counselling are the most relevant strategies for mothers and their children. The use of antioxidants and/or NRT without stopping the smoking habit can increase the nicotine burden on fetal and neonatal lungs and encourage the mother to continue nicotine dependence and transfer it to the next generations. Community-based programs that involve partners, family members, and social networks can create a supportive environment for behaviour change. Similarly, collaborations between healthcare systems, public health agencies, and advocacy groups can amplify the reach and effectiveness of prevention efforts.

## References

[1] La Grutta S., Indinnimeo L., di Coste A., Ferrante G., Landi M., Pelosi U., Rusconi F. (2013). Environmental Risk Factors and Lung Diseases in Children: From Guidelines to Health Effects. Early Hum. Dev..

[2] Palazzolo D.L. (2013). Electronic Cigarettes and Vaping: A New Challenge in Clinical Medicine and Public Health. A Literature Review. Front. Public Health.

[3] McRobbie H., Bullen C., Hartmann-Boyce J., Hajek P., McRobbie H. (2014). Electronic Cigarettes for Smoking Cessation and Reduction. Cochrane Database of Systematic Reviews.

[4] Yoong S.L., Hall A., Turon H., Stockings E., Leonard A., Grady A., Tzelepis F., Wiggers J., Gouda H., Fayokun R. (2021). Association between Electronic Nicotine Delivery Systems and Electronic Non-Nicotine Delivery Systems with Initiation of Tobacco Use in Individuals Aged < 20 Years. A Systematic Review and Meta-Analysis. PLoS ONE.

[5] Breitbarth A.K., Morgan J., Jones A.L. (2018). E-Cigarettes—An Unintended Illicit Drug Delivery System. Drug Alcohol. Depend..

[6] Sosnowski T.R., Odziomek M. (2018). Particle Size Dynamics: Toward a Better Understanding of Electronic Cigarette Aerosol Interactions With the Respiratory System. Front. Physiol..

[7] Stefaniak A.B., LeBouf R.F., Ranpara A.C., Leonard S.S. (2021). Toxicology of Flavoring- and Cannabis-Containing e-Liquids Used in Electronic Delivery Systems. Pharmacol. Ther..

[8] Stroud L.R., Papandonatos G.D., Borba K., Kehoe T., Scott-Sheldon L.A.J. (2019). Flavored Electronic Cigarette Use, Preferences, and Perceptions in Pregnant Mothers: A Correspondence Analysis Approach. Addict. Behav..

[9] Goniewicz M.L., Knysak J., Gawron M., Kosmider L., Sobczak A., Kurek J., Prokopowicz A., Jablonska-Czapla M., Rosik-Dulewska C., Havel C. (2014). Levels of Selected Carcinogens and Toxicants in Vapour from Electronic Cigarettes. Tob. Control.

[10] Palazzolo D.L., Crow A.P., Nelson J.M., Johnson R.A. (2017). Trace Metals Derived from Electronic Cigarette (ECIG) Generated Aerosol: Potential Problem of ECIG Devices That Contain Nickel. Front. Physiol..

[11] Jensen R.P., Luo W., Pankow J.F., Strongin R.M., Peyton D.H. (2015). Hidden Formaldehyde in E-Cigarette Aerosols. N. Engl. J. Med..

[12] Mark K.S., Farquhar B., Chisolm M.S., Coleman-Cowger V.H., Terplan M. (2015). Knowledge, Attitudes, and Practice of Electronic Cigarette Use Among Pregnant Women. J. Addict. Med..

[13] Ashford K., Wiggins A., Butler K., Ickes M., Rayens M.K., Hahn E. (2016). E-Cigarette Use and Perceived Harm Among Women of Childbearing Age Who Reported Tobacco Use During the Past Year. Nurs. Res..

[14] Reynolds C.M.E., Egan B., McKeating A., Daly N., Sheehan S.R., Turner M.J. (2017). Five Year Trends in Maternal Smoking Behaviour Reported at the First Prenatal Appointment. Ir. J. Med. Sci..

[15] Whittington J.R., Simmons P.M., Phillips A.M., Gammill S.K., Cen R., Magann E.F., Cardenas V.M. (2018). The Use of Electronic Cigarettes in Pregnancy: A Review of the Literature. Obs. Gynecol. Surv..

[16] Wagner N.J., Camerota M., Propper C. (2017). Prevalence and Perceptions of Electronic Cigarette Use during Pregnancy. Matern. Child. Health J..

[17] McCubbin A., Fallin-Bennett A., Barnett J., Ashford K. (2017). Perceptions and Use of Electronic Cigarettes in Pregnancy. Health Educ. Res..

[18] Kuniyoshi K.M., Rehan V.K. (2019). The Impact of Perinatal Nicotine Exposure on Fetal Lung Development and Subsequent Respiratory Morbidity. Birth Defects Res..

[19] Orzabal M.R., Naik V.D., Lee J., Hillhouse A.E., Brashear W.A., Threadgill D.W., Ramadoss J. (2022). Impact of E-Cig Aerosol Vaping on Fetal and Neonatal Respiratory Development and Function. Transl. Res..

[20] McEvoy C.T., Spindel E.R. (2017). Pulmonary Effects of Maternal Smoking on the Fetus and Child: Effects on Lung Development, Respiratory Morbidities, and Life Long Lung Health. Paediatr. Respir. Rev..

[21] Wills T.A., Soneji S.S., Choi K., Jaspers I., Tam E.K. (2021). E-Cigarette Use and Respiratory Disorders: An Integrative Review of Converging Evidence from Epidemiological and Laboratory Studies. Eur. Respir. J..

[22] Calogero C., Sly P.D. (2010). Developmental Physiology: Lung Function during Growth and Development from Birth to Old Age. Paediatric Lung Function.

[23] Rehman S., Bacha D. (2025). Embryology, Pulmonary.

[24] Schittny J.C. (2017). Development of the Lung. Cell Tissue Res..

[25] Koos B.J., Rajaee A. (2014). Fetal Breathing Movements and Changes at Birth.

[26] Pan J., Copland I., Post M., Yeger H., Cutz E. (2006). Mechanical Stretch-Induced Serotonin Release from Pulmonary Neuroendocrine Cells: Implications for Lung Development. Am. J. Physiol.-Lung Cell Mol. Physiol..

[27] Dunnill M.S. (1962). Postnatal Growth of the Lung. Thorax.

[28] Thurlbeck W.M. (1982). Postnatal Human Lung Growth. Thorax.

[29] Lovric G., Barré S.F., Schittny J.C., Roth-Kleiner M., Stampanoni M., Mokso R. (2013). Dose Optimization Approach to Fast X-Ray Microtomography of the Lung Alveoli. J. Appl. Crystallogr..

[30] Vasilescu D.M., Gao Z., Saha P.K., Yin L., Wang G., Haefeli-Bleuer B., Ochs M., Weibel E.R., Hoffman E.A. (2012). Assessment of Morphometry of Pulmonary Acini in Mouse Lungs by Nondestructive Imaging Using Multiscale Microcomputed Tomography. Proc. Natl. Acad. Sci. USA.

[31] Yablonskiy D.A., Sukstanskii A.L., Woods J.C., Gierada D.S., Quirk J.D., Hogg J.C., Cooper J.D., Conradi M.S. (2009). Quantification of Lung Microstructure with Hyperpolarized ^3^He Diffusion MRI. J. Appl. Physiol..

[32] Hislop A., Muir D.C.F., Jacobsen M., Simon G., Reid L. (1972). Postnatal Growth and Function of the Pre-Acinar Airways. Thorax.

[33] Selevan S., Kimmel C., Mendola P. (2015). Windows of Susceptibility to Environmental Exposures in Children: (Excerpt from Children’s Health and the Environment: A Global Perspective). Everyday Environmental Toxins.

[34] Kovesi T., Rubin S. (2004). Long-Term Complications of Congenital Esophageal Atresia and/or Tracheoesophageal Fistula. Chest.

[35] Kool H., Mous D., Tibboel D., de Klein A., Rottier R.J. (2014). Pulmonary Vascular Development Goes Awry in Congenital Lung Abnormalities. Birth Defects Res. Part. C Embryo Today.

[36] Phalen R.F., Oldham M.J., Beaucage C.B., Crocker T.T., Mortensen J. (1985). Postnatal Enlargement of Human Tracheobronchial Airways and Implications for Particle Deposition. Anat. Rec..

[37] Wen X., Liu L., Moe A.A., Ormond I.K., Shuren C.C., Scott I.N., Ozga J.E., Stanton C.A., Ruybal A.L., Hart J.L. (2023). Use of E-Cigarettes and Cigarettes During Late Pregnancy Among Adolescents. JAMA Netw. Open.

[38] Mescolo F., Ferrante G., La Grutta S. (2021). Effects of E-Cigarette Exposure on Prenatal Life and Childhood Respiratory Health: A Review of Current Evidence. Front. Pediatr..

[39] Prochaska J.J., Vogel E.A., Benowitz N. (2022). Nicotine Delivery and Cigarette Equivalents from Vaping a JUULpod. Tob. Control.

[40] Luck W., Nau H., Hansen R., Steldinger R. (1985). Extent of Nicotine and Cotinine Transfer to the Human Fetus, Placenta and Amniotic Fluid of Smoking Mothers. Dev. Pharmacol. Ther..

[41] Napierala M., Mazela J., Merritt T.A., Florek E. (2016). Tobacco Smoking and Breastfeeding: Effect on the Lactation Process, Breast Milk Composition and Infant Development. A Critical Review. Environ. Res..

[42] Rowe H., Baker T., Hale T.W. (2015). Maternal Medication, Drug Use, and Breastfeeding. Child. Adolesc. Psychiatr. Clin. N. Am..

[43] Suter M.A., Mastrobattista J., Sachs M., Aagaard K. (2015). Is There Evidence for Potential Harm of Electronic Cigarette Use in Pregnancy?. Birth Defects Res. A Clin. Mol. Teratol..

[44] Rao P., Liu J., Springer M.L. (2020). JUUL and Combusted Cigarettes Comparably Impair Endothelial Function. Tob. Regul. Sci..

[45] St. Helen G., Havel C., Dempsey D.A., Jacob P., Benowitz N.L. (2016). Nicotine Delivery, Retention and Pharmacokinetics from Various Electronic Cigarettes. Addiction.

[46] Ozekin Y.H., Saal M.L., Pineda R.H., Moehn K., Ordonez-Erives M.A., Delgado Figueroa M.F., Frazier C., Korth K.M., Königshoff M., Bates E.A. (2023). Intrauterine Exposure to Nicotine through Maternal Vaping Disrupts Embryonic Lung and Skeletal Development via the Kcnj2 Potassium Channel. Dev. Biol..

[47] Noël A., Hansen S., Zaman A., Perveen Z., Pinkston R., Hossain E., Xiao R., Penn A. (2020). In Utero Exposures to Electronic-Cigarette Aerosols Impair the *Wnt* Signaling during Mouse Lung Development. Am. J. Physiol.-Lung Cell. Mol. Physiol..

[48] Beers M.F., Morrisey E.E. (2011). The Three R’s of Lung Health and Disease: Repair, Remodeling, and Regeneration. J. Clin. Investig..

[49] Tilley A.E., Harvey B.-G., Heguy A., Hackett N.R., Wang R., O’Connor T.P., Crystal R.G. (2009). Down-Regulation of the Notch Pathway in Human Airway Epithelium in Association with Smoking and Chronic Obstructive Pulmonary Disease. Am. J. Respir. Crit. Care Med..

[50] Hussain M., Xu C., Lu M., Wu X., Tang L., Wu X. (2017). Wnt/β-Catenin Signaling Links Embryonic Lung Development and Asthmatic Airway Remodeling. Biochim. Biophys. Acta (BBA)—Mol. Basis Dis..

[51] McGrath-Morrow S.A., Hayashi M., Aherrera A., Lopez A., Malinina A., Collaco J.M., Neptune E., Klein J.D., Winickoff J.P., Breysse P. (2015). The Effects of Electronic Cigarette Emissions on Systemic Cotinine Levels, Weight and Postnatal Lung Growth in Neonatal Mice. PLoS ONE.

[52] Gambadauro A., Galletta F., Li Pomi A., Manti S., Piedimonte G. (2024). Immune Response to Respiratory Viral Infections. Int. J. Mol. Sci..

[53] Palazzolo D.L., Nelson J.M., Ely E.A., Crow A.P., Distin J., Kunigelis S.C. (2017). The Effects of Electronic Cigarette (ECIG)-Generated Aerosol and Conventional Cigarette Smoke on the Mucociliary Transport Velocity (MTV) Using the Bullfrog (*R. catesbiana*) Palate Paradigm. Front. Physiol..

[54] Laube B.L., Afshar-Mohajer N., Koehler K., Chen G., Lazarus P., Collaco J.M., McGrath-Morrow S.A. (2017). Acute and Chronic in Vivo Effects of Exposure to Nicotine and Propylene Glycol from an E-Cigarette on Mucociliary Clearance in a Murine Model. Inhal. Toxicol..

[55] Chung S., Baumlin N., Dennis J.S., Moore R., Salathe S.F., Whitney P.L., Sabater J., Abraham W.M., Kim M.D., Salathe M. (2019). Electronic Cigarette Vapor with Nicotine Causes Airway Mucociliary Dysfunction Preferentially via TRPA1 Receptors. Am. J. Respir. Crit. Care Med..

[56] Manti S., Gambadauro A., Galletta F., Ruggeri P., Piedimonte G. (2024). Update on the Role of Β2AR and TRPV1 in Respiratory Diseases. Int. J. Mol. Sci..

[57] Ogunwale M.A., Li M., Ramakrishnam Raju M.V., Chen Y., Nantz M.H., Conklin D.J., Fu X.-A. (2017). Aldehyde Detection in Electronic Cigarette Aerosols. ACS Omega.

[58] Sinharoy P., McAllister S.L., Vasu M., Gross E.R. (2019). Environmental Aldehyde Sources and the Health Implications of Exposure.

[59] Kim M.D., Chung S., Dennis J.S., Yoshida M., Aguiar C., Aller S.P., Mendes E.S., Schmid A., Sabater J., Baumlin N. (2022). Vegetable Glycerin E-Cigarette Aerosols Cause Airway Inflammation and Ion Channel Dysfunction. Front. Pharmacol..

[60] Kim M.D., Chung S., Baumlin N., Qian J., Montgomery R.N., Sabater J., Berkland C., Salathe M. (2024). The Combination of Propylene Glycol and Vegetable Glycerin E-Cigarette Aerosols Induces Airway Inflammation and Mucus Hyperconcentration. Sci. Rep..

[61] Aslaner D.M., Alghothani O., Saldana T.A., Ezell K.G., Yallourakis M.D., MacKenzie D.M., Miller R.A., Wold L.E., Gorr M.W. (2022). E-Cigarette Vapor Exposure in Utero Causes Long-Term Pulmonary Effects in Offspring. Am. J. Physiol.-Lung Cell. Mol. Physiol..

[62] Wang Q., Sundar I.K., Blum J.L., Ratner J.R., Lucas J.H., Chuang T.-D., Wang Y., Liu J., Rehan V.K., Zelikoff J.T. (2020). Prenatal Exposure to Electronic-Cigarette Aerosols Leads to Sex-Dependent Pulmonary Extracellular-Matrix Remodeling and Myogenesis in Offspring Mice. Am. J. Respir. Cell Mol. Biol..

[63] Lucas J.H., Muthumalage T., Wang Q., Friedman M.R., Friedman A.E., Rahman I. (2020). E-Liquid Containing a Mixture of Coconut, Vanilla, and Cookie Flavors Causes Cellular Senescence and Dysregulated Repair in Pulmonary Fibroblasts: Implications on Premature Aging. Front. Physiol..

[64] Hua M., Omaiye E.E., Luo W., McWhirter K.J., Pankow J.F., Talbot P. (2019). Identification of Cytotoxic Flavor Chemicals in Top-Selling Electronic Cigarette Refill Fluids. Sci. Rep..

[65] Behar R.Z., Davis B., Wang Y., Bahl V., Lin S., Talbot P. (2014). Identification of Toxicants in Cinnamon-Flavored Electronic Cigarette Refill Fluids. Toxicol. Vitr..

[66] Ma T., Wang X., Li L., Sun B., Zhu Y., Xia T. (2021). Electronic Cigarette Aerosols Induce Oxidative Stress-Dependent Cell Death and NF-ΚB Mediated Acute Lung Inflammation in Mice. Arch. Toxicol..

[67] Hecker L. (2018). Mechanisms and Consequences of Oxidative Stress in Lung Disease: Therapeutic Implications for an Aging Populace. Am. J. Physiol.-Lung Cell Mol. Physiol..

[68] Emma R., Caruso M., Campagna D., Pulvirenti R., Li Volti G. (2022). The Impact of Tobacco Cigarettes, Vaping Products and Tobacco Heating Products on Oxidative Stress. Antioxidants.

[69] Sussan T.E., Gajghate S., Thimmulappa R.K., Ma J., Kim J.-H., Sudini K., Consolini N., Cormier S.A., Lomnicki S., Hasan F. (2015). Exposure to Electronic Cigarettes Impairs Pulmonary Anti-Bacterial and Anti-Viral Defenses in a Mouse Model. PLoS ONE.

[70] Chen H., Li G., Chan Y.L., Chapman D.G., Sukjamnong S., Nguyen T., Annissa T., McGrath K.C., Sharma P., Oliver B.G. (2018). Maternal E-Cigarette Exposure in Mice Alters DNA Methylation and Lung Cytokine Expression in Offspring. Am. J. Respir. Cell Mol. Biol..

[71] Lerner C.A., Sundar I.K., Yao H., Gerloff J., Ossip D.J., McIntosh S., Robinson R., Rahman I. (2015). Vapors Produced by Electronic Cigarettes and E-Juices with Flavorings Induce Toxicity, Oxidative Stress, and Inflammatory Response in Lung Epithelial Cells and in Mouse Lung. PLoS ONE.

[72] Abusara O.H., Hammad A.M., Debas R., Al-Shalabi E., Waleed M., Scott Hall F. (2025). The Inflammation and Oxidative Status of Rat Lung Tissue Following Smoke/Vapor Exposure via E-Cigarette, Cigarette, and Waterpipe. Gene.

[73] El-Mahdy M.A., Mahgoup E.M., Ewees M.G., Eid M.S., Abdelghany T.M., Zweier J.L. (2021). Long-Term Electronic Cigarette Exposure Induces Cardiovascular Dysfunction Similar to Tobacco Cigarettes: Role of Nicotine and Exposure Duration. Am. J. Physiol.-Heart Circ. Physiol..

[74] Moheimani R.S., Bhetraratana M., Yin F., Peters K.M., Gornbein J., Araujo J.A., Middlekauff H.R. (2017). Increased Cardiac Sympathetic Activity and Oxidative Stress in Habitual Electronic Cigarette Users. JAMA Cardiol..

[75] El-Mahdy M.A., Ewees M.G., Eid M.S., Mahgoup E.M., Khaleel S.A., Zweier J.L. (2022). Electronic Cigarette Exposure Causes Vascular Endothelial Dysfunction Due to NADPH Oxidase Activation and ENOS Uncoupling. Am. J. Physiol.-Heart Circ. Physiol..

[76] Neuman Å., Hohmann C., Orsini N., Pershagen G., Eller E., Kjaer H.F., Gehring U., Granell R., Henderson J., Heinrich J. (2012). Maternal Smoking in Pregnancy and Asthma in Preschool Children. Am. J. Respir. Crit. Care Med..

[77] Burke H., Leonardi-Bee J., Hashim A., Pine-Abata H., Chen Y., Cook D.G., Britton J.R., McKeever T.M. (2012). Prenatal and Passive Smoke Exposure and Incidence of Asthma and Wheeze: Systematic Review and Meta-Analysis. Pediatrics.

[78] Zaitsu M., Kono K., Hosokawa Y., Miyamoto M., Nanishi K., Okawa S., Niki S., Takahashi K., Yoshihara S., Kobashi G. (2023). Maternal Heated Tobacco Product Use during Pregnancy and Allergy in Offspring. Allergy.

[79] Martinez F.D., Wright A.L., Taussig L.M., Holberg C.J., Halonen M., Morgan W.J. (1995). Asthma and Wheezing in the First Six Years of Life. N. Engl. J. Med..

[80] Lodrup Carlsen K., Jaakkola J., Nafstad P., Carlsen K. (1997). In Utero Exposure to Cigarette Smoking Influences Lung Function at Birth. Eur. Respir. J..

[81] Gotts J.E., Jordt S.-E., McConnell R., Tarran R. (2019). What Are the Respiratory Effects of E-Cigarettes?. BMJ.

[82] Costantino S., Torre A., Foti Randazzese S., Mollica S.A., Motta F., Busceti D., Ferrante F., Caminiti L., Crisafulli G., Manti S. (2024). Association between Second-Hand Exposure to E-Cigarettes at Home and Exacerbations in Children with Asthma. Children.

[83] Wills T.A., Pagano I., Williams R.J., Tam E.K. (2019). E-Cigarette Use and Respiratory Disorder in an Adult Sample. Drug Alcohol. Depend..

[84] Bailey C., Medeiros P.d.B., Ellwood D.A., Middleton P., Andrews C.J., Flenady V.J. (2023). A Systematic Review of Interventions to Increase the Use of Smoking Cessation Services for Women Who Smoke during Pregnancy. Aust. N. Z. J. Obstet. Gynaecol..

[85] Diamanti A., Papadakis S., Schoretsaniti S., Rovina N., Vivilaki V., Gratziou C., Katsaounou P. (2019). Smoking Cessation in Pregnancy: An Update for Maternity Care Practitioners. Tob. Induc. Dis..

[86] Bérard A., Zhao J.-P., Sheehy O. (2016). Success of Smoking Cessation Interventions during Pregnancy. Am. J. Obs. Gynecol..

[87] Madureira J., Camelo A., Silva A.I., Reis A.T., Esteves F., Ribeiro A.I., Teixeira J.P., Costa C. (2020). The Importance of Socioeconomic Position in Smoking, Cessation and Environmental Tobacco Smoke Exposure during Pregnancy. Sci. Rep..

[88] Chamberlain C., O’Mara-Eves A., Porter J., Coleman T., Perlen S.M., Thomas J., McKenzie J.E. (2017). Psychosocial Interventions for Supporting Women to Stop Smoking in Pregnancy. Cochrane Database Syst. Rev..

[89] Ussher M., Lewis S., Aveyard P., Manyonda I., West R., Lewis B., Marcus B., Riaz M., Taylor A., Daley A. (2015). Physical Activity for Smoking Cessation in Pregnancy: Randomised Controlled Trial. BMJ.

[90] Georgakopoulou V.E., Diamanti A. (2024). Artificial Intelligence for Smoking Cessation in Pregnancy. Cureus.

[91] McMeekin N., Sinclair L., Robinson-Smith L., Mitchell A., Bauld L., Tappin D.M., Boyd K.A. (2023). Financial Incentives for Quitting Smoking in Pregnancy: Are They Cost-effective?. Addiction.

[92] Boyd K.A., Briggs A.H., Bauld L., Sinclair L., Tappin D. (2016). Are Financial Incentives Cost-Effective to Support Smoking Cessation during Pregnancy?. Addiction.

[93] Tappin D., Bauld L., Purves D., Boyd K., Sinclair L., MacAskill S., McKell J., Friel B., McConnachie A., de Caestecker L. (2015). Financial Incentives for Smoking Cessation in Pregnancy: Randomised Controlled Trial. BMJ.

[94] Scherman A., Tolosa J.E., McEvoy C. (2018). Smoking Cessation in Pregnancy: A Continuing Challenge in the United States. Ther. Adv. Drug Saf..

[95] Vila-Farinas A., Pérez-Rios M., Montes-Martinez A., Ruano-Ravina A., Forray A., Rey-Brandariz J., Candal-Pedreira C., Fernández E., Casal-Acción B., Varela-Lema L. (2024). Effectiveness of Smoking Cessation Interventions among Pregnant Women: An Updated Systematic Review and Meta-Analysis. Addict. Behav..

[96] Gould G.S., Twyman L., Stevenson L., Gribbin G.R., Bonevski B., Palazzi K., Bar Zeev Y. (2019). What Components of Smoking Cessation Care during Pregnancy Are Implemented by Health Providers? A Systematic Review and Meta-Analysis. BMJ Open.

[97] Blanc J., Tosello B., Ekblad M.O., Berlin I., Netter A. (2021). Nicotine Replacement Therapy during Pregnancy and Child Health Outcomes: A Systematic Review. Int. J. Environ. Res. Public Health.

[98] Hartmann-Boyce J., Chepkin S.C., Ye W., Bullen C., Lancaster T. (2018). Nicotine Replacement Therapy versus Control for Smoking Cessation. Cochrane Database Syst. Rev..

[99] Claire R., Chamberlain C., Davey M.-A., Cooper S.E., Berlin I., Leonardi-Bee J., Coleman T. (2020). Pharmacological Interventions for Promoting Smoking Cessation during Pregnancy. Cochrane Database Syst. Rev..

[100] Robijn A.L., Tran D.T., Cohen J.M., Donald S., Cesta C.E., Furu K., Parkin L., Pearson S.-A., Reutfors J., Zoega H. (2024). Smoking Cessation Pharmacotherapy Use in Pregnancy. JAMA Netw. Open.

[101] Tran D.T., Preen D.B., Einarsdottir K., Kemp-Casey A., Randall D., Jorm L.R., Choi S.K.Y., Havard A. (2020). Use of Smoking Cessation Pharmacotherapies during Pregnancy Is Not Associated with Increased Risk of Adverse Pregnancy Outcomes: A Population-Based Cohort Study. BMC Med..

[102] Morales-Suárez-Varela M., Puig B.M., Kaerlev L., Peraita-Costa I., Perales-Marín A. (2022). Safety of Nicotine Replacement Therapy during Pregnancy: A Narrative Review. Int. J. Environ. Res. Public Health.

[103] Gambadauro A., Foti Randazzese S., Currò A., Galletta F., Crisafulli G., Caminiti L., Germanò E., Di Rosa G., Nicotera A.G., Manti S. (2023). Impact of the Allergic Therapeutic Adherence in Children with Allergic Rhinitis and ADHD: A Pilot Study. J. Pers. Med..

[104] Barboza J. (2018). Pharmaceutical Strategies for Smoking Cessation during Pregnancy. Expert. Opin. Pharmacother..

[105] Adams E.K., Markowitz S., Kannan V., Dietz P.M., Tong V.T., Malarcher A.M. (2012). Reducing Prenatal Smoking. Am. J. Prev. Med..

[106] McEvoy C.T., Schilling D., Clay N., Jackson K., Go M.D., Spitale P., Bunten C., Leiva M., Gonzales D., Hollister-Smith J. (2014). Vitamin C Supplementation for Pregnant Smoking Women and Pulmonary Function in Their Newborn Infants. JAMA.

[107] Shorey-Kendrick L.E., McEvoy C.T., Ferguson B., Burchard J., Park B.S., Gao L., Vuylsteke B.H., Milner K.F., Morris C.D., Spindel E.R. (2017). Vitamin C Prevents Offspring DNA Methylation Changes Associated with Maternal Smoking in Pregnancy. Am. J. Respir. Crit. Care Med..

[108] Shorey-Kendrick L.E., McEvoy C.T., O’Sullivan S.M., Milner K., Vuylsteke B., Tepper R.S., Morgan T.K., Roberts V.H.J., Lo J.O., Frias A.E. (2024). Vitamin C Supplementation Improves Placental Function and Alters Placental Gene Expression in Smokers. Sci. Rep..

[109] McEvoy C.T., Shorey-Kendrick L.E., Milner K., Schilling D., Tiller C., Vuylsteke B., Scherman A., Jackson K., Haas D.M., Harris J. (2019). Oral Vitamin C (500 Mg/d) to Pregnant Smokers Improves Infant Airway Function at 3 Months (VCSIP). A Randomized Trial. Am. J. Respir. Crit. Care Med..

[110] Gallo C., Renzi P., Loizzo S., Loizzo A., Piacente S., Festa M., Caputo M., Tecce M.F., Capasso A. (2010). Potential Therapeutic Effects of Vitamin E and C on Placental Oxidative Stress Induced by Nicotine: An In Vitro Evidence. Open Biochem. J..

[111] Wang B., Chan Y.L., Zhou S., Saad S., Chen H., Oliver B.G. (2020). Offspring Sex Affects the Susceptibility to Maternal Smoking-Induced Lung Inflammation and the Effect of Maternal Antioxidant Supplementation in Mice. J. Inflamm..

